# Case clustering, contact stratification, and transmission heterogeneity of SARS-CoV-2 Omicron BA.5 variants in Urumqi, China: An observational study

**DOI:** 10.7189/jogh.13.06018

**Published:** 2023-05-19

**Authors:** Yaoqin Lu, Zihao Guo, Ting Zeng, Shengzhi Sun, Yanmei Lu, Zhidong Teng, Maozai Tian, Jun Wang, Shulin Li, Xucheng Fan, Zemin Luan, Weiming Wang, Yongli Cai, Kai Wang, Shi Zhao

**Affiliations:** 1 School of Public Health, Xinjiang Medical University, Urumqi, China; 2 Urumqi Center for Disease Control and Prevention, Urumqi, China; 3 JC School of Public Health and Primary Care, Chinese University of Hong Kong, Hong Kong, China; 4 Department of Medical Engineering and Technology, Xinjiang Medical University, Urumqi, China; 5 Department of Epidemiology and Biostatistics, School of Public Health, Capital Medical University, Beijing, China; 6 Department of Cardiac Pacing and Electrophysiology, The First Affiliated Hospital of Xinjiang Medical University, Urumqi, China; 7 School of Mathematics and Statistics, Huaiyin Normal University, Huaian, China; 8 Centre for Health Systems and Policy Research, Chinese University of Hong Kong, Hong Kong, China

## Abstract

**Background:**

From August to September 2022, Urumqi, the capital of the Xinjiang Uygur Autonomous Region in China, faced its largest COVID-19 outbreak caused by the emergence of the SARS-CoV-2 Omicron BA.5.2 variants. Although the superspreading of COVID-19 played an important role in triggering large-scale outbreaks, little was known about the superspreading potential and heterogeneity in the transmission of Omicron BA.5 variants.

**Methods:**

In this retrospective observational, contact tracing study, we identified 1139 laboratory-confirmed COVID-19 cases of Omicron BA.5.2 variants, and 51 323 test-negative close contacts in Urumqi from 7 August to 7 September 2022. By using detailed contact tracing information and exposure history of linked case-contact pairs, we described stratification in contact and heterogeneity in transmission across different demographic strata, vaccine statuses, and contact settings. We adopted beta-binomial models to characterise the secondary attack rate (SAR) distribution among close contacts and modelled COVID-19 transmission as a branching process with heterogeneity in transmission governed by negative binomial models.

**Results:**

After the city lockdown, the mean case cluster size decreased from 2.0 (before lockdown) to 1.6, with decreased proportions of contacts in workplace and community settings compared with household settings. We estimated that 14% of the most infectious index cases generated 80% transmission, whereas transmission in the community setting presented the highest heterogeneity, with 5% index cases seeding 80% transmission. Compared with zero, one, and two doses of inactivated vaccine (Sinopharm), index cases with three doses of vaccine had a lower risk of generating secondary cases in terms of the reproduction number. Contacts of female cases, cases with ages 0-17 years, and household settings had relatively higher SAR.

**Conclusions:**

In the context of intensive control measures, active case detection, and relatively high vaccine coverage, but with an infection-naive population, our findings suggested high heterogeneity in the contact and transmission risks of Omicron BA.5 variants across different demographic strata, vaccine statuses, and contact settings. Given the rapid evolution of SARS-CoV-2, investigating the distribution of transmission not only helped promote public awareness and preparedness among high-risk groups, but also highlighted the importance of continuously monitoring the transmission characteristics of genetic variants of SARS-CoV-2.

The global COVID-19 pandemic has been sustained by the SARS-CoV-2 Omicron variant, which is the fifth variant of concern (VOC) declared by the World Health Organization (WHO) in November 2021 [1]. The pandemic was dominated by the Omicron BA.5 variants (first detected in South Africa in February 2022) which, along with its descendants, accounted for more than 78.9% of all viral sequences sampled globally during epidemiological week 39 in 2022 (September 26 to October 2, 2022) [2].

Preventing and controlling the VOC outbreaks is often challenging due to the evolved characteristics compared with historical strains [3-5] While vaccination has been rapidly ramped up in most of the regions, up-to-date evidence suggests its waned effectiveness against the Omicron BA.5 variant due to its significant ability of immune escaping [6,7]. Public health and social measures (PHSMs), such as contact tracing, case isolation, and region-wide lockdown, have been effective for rapid outbreak control [8,9]. However, there is often concern about superspreading events (defined as the single-generation spread of infection that involves an unusually large number of cases), as they could lead to large outbreaks despite strict PHSMs [10-14]. Such events may arise from heterogeneous outbreak dynamics, where most transmissions were generated by a small fraction of cases that may be biologically (e.g. higher within-host viral load) and/or behaviourally (e.g. had more social interaction with other people) different from other infected cases [15,16]. COVID-19 outbreaks are often heterogeneous. Studies that characterised the transmission heterogeneity of the COVID-19 pandemic suggested that 80% of the transmissions were seeded by 19% of the cases for the wild-type strains 11, as opposed to 15% for the Delta variants (the previous global circulating VOC) and 9% for the Omicron BA.1 variant [17]. Assessing the transmission heterogeneity for an infectious agent in context could aid in understanding disease transmissibility.

According to classic epidemiological theory [18], virus transmissibility can be described by an effective reproduction number *R* (defined as the average number of cases an infectious case can generate in a particular time) that represents the average transmissibility of a virus, and a dispersion parameter *k* that reflects the individual heterogeneity in *R*. The heterogeneity in *R* could arise from individual differences in the social contact pattern [19,20] (e.g. settings where contact occurred, number of contacts made), the duration of the infectious period [21], and the probability of infection being transmitted per contact that can be quantified by the secondary attack rate (SAR) [22]. Previous studies suggested that contact, secondary transmission, and SAR during the early phase of the COVID-19 epidemic differed substantially by demographic factors (e.g. age and sex), contact settings, and clinical severity [19,20,23,24].

Targeted PHSMs that were indicated by heterogeneous transmission risks and contact patterns could give rise to the efficient control of outbreaks [10,25-27]. To date, little is known about the heterogeneity in the transmission of the VOC, especially for the current circulating Omicron BA.5. [27]. Using detailed contact tracing data collected from Urumqi, an epicentre of the Omicron BA.5.2 outbreak in Xinjiang, China, we assessed the stratification in contact patterns and variation in the transmission risks to examine the level of heterogeneity in transmission and superspreading potentials of the Omicron BA.5 variants.

## METHODS

### Study design and setting

This was a retrospective observational, contact tracing study including all laboratory-confirmed COVID-19 cases and close contacts identified from 7 August to 7 September 2022, in Urumqi, China, with information on exposure risks by linking case-contact pairs.

As of July 2022, the vaccination coverage was 90% and >72% of two and three doses of inactivated (Sinopharm) vaccines, respectively, among the general population of mainland China, similar to that among the inhabitants of Urumqi city. Since mainland China implemented the “zero COVID-19” policy in 2020, no large-scale COVID-19 outbreak had occurred in Urumqi or in most of China.

The first COVID-19 case infected by the Omicron BA.5.2 variant was detected in Urumqi on 7 August 2022. Since then, the variant spread promptly and the epidemic peaked on 13 August 2022. In response, the local government imposed intense temporary “static management” measures on 10 August, including a city-wide lockdown, mass testing, symptom-based surveillance, contact tracing, and case isolation [28]. All confirmed cases were sent to the appointed hospital, where detailed epidemiological investigations for each case were conducted to record their exposure and contact history. The identified close contacts of the confirmed cases were immediately sent to the quarantine centre for routine real-time reverse transcription polymerase chain reaction (RT-PCR) testing and medical observation. Additionally, all individuals in Urumqi underwent RT-PCR testing on a daily basis (i.e. city-wide mass detection) to proactively detect COVID-19 cases. The “zero COVID-19” measures remained active on mainland China until 28 November 2022. We investigated the characteristics of SARS-CoV-2 Omicron transmission during the “zero COVID-19” period.

### Participants

We collected epidemiological contact tracing data for Omicron BA.5.2 cases reported between 7 August and 7 September 2022, from the Centre for Disease Control and Prevention in Urumqi, China. All cases were laboratory-confirmed by RT-PCR on a nasopharyngeal or oropharyngeal swab (Appendix S1 in the **Online Supplementary Document**). For each case, we extracted information on age, sex, contact and exposure history, contact settings (i.e. household, community, workplace, and unknown contact settings), symptom onset date, case diagnosis date, and vaccination history. We excluded cases without available patient record information. Contact tracing data were collected and analysed as part of an ongoing public health outbreak investigation. All confirmed cases were sent to designated hospitals, where each case underwent rigorous epidemiological investigation by recording contact and exposure history. All close contacts of confirmed COVID-19 cases before diagnosis were immediately quarantined for 10 days, during which symptom (e.g. continuous fever, cough) monitoring, and RT-PCR tests were conducted for all citizens on a daily basis. According to the local COVID-19 control measures policy, daily RT-PCR tests were mandatory for all residents in Urumqi. Test-negative close contacts without influenza-like symptoms were released after a 10-day quarantine period.

The SARS-CoV-2 genetic samples of 11 COVID-19 cases confirmed in the first few days of the outbreaks were sent for whole genome sequencing. The SARS-CoV-2 genetic variants that caused this outbreak were classified as the Omicron BA.5.2 sub-lineage per the PANGO lineage designation (Appendix S2 in the **Online Supplementary Document**).

We defined close contacts as individuals who had close contact records with laboratory-confirmed COVID-19 patients. We categorised the identified close contacts based on the corresponding contact settings, including the household, community, workplaces, and unknown settings (i.e. the information on the type of settings was not available). We identified epidemiological links between cases and constructed infector-infectee transmission pairs per the contact and exposure history of individual cases, defining a group of cases that are epidemiologically linked with a common single source of infection as a case cluster. A case cluster only involved a single generation, although there could be linkages between case clusters (i.e. transmission chain). Sporadic cases (cases leading to no secondary cases) were counted as case clusters with a size equal to one. The details of the identification of transmission pairs are provided in Appendix S3 in the **Online Supplementary Document**.

### Statistical analyses

**Figure 1:**
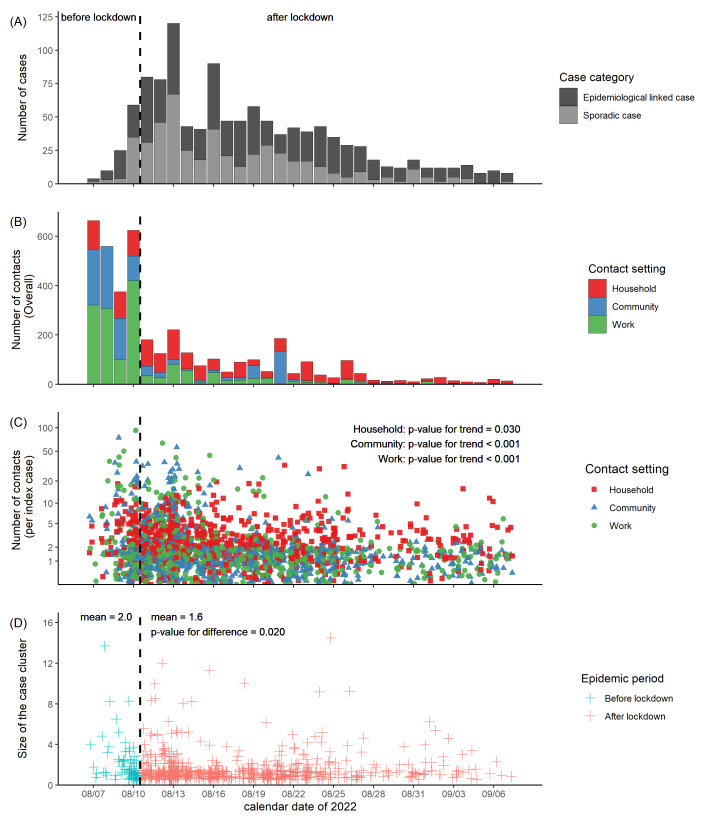
The epidemic curve, the trend of number of close contacts, and the case cluster size from 7 August to 7 September 2022 in Urumqi, China. **Panel A.** Epidemic curve of the daily number of laboratory-confirmed COVID-19 cases by the test-positive date. **Panel B.** Daily number of close contacts by the last date of contact with their associated index cases. **Panel C.** Number of close contacts of each index case by the last date of contact, stratified by contact settings. **Panel D.** The case cluster size by the test-positive date of the identified seed case of the cluster, stratified by epidemic periods before vs after city lockdown (the vertical bold dashed line).

We summarised close contact characteristics and case cluster sizes using descriptive statistics and used generalised linear regression models to analyse the temporal trend of contact frequencies. The model described in **Figure 1**, panel C used a general linear model (GLM), with the number of contacts as the dependent and the calendar date as the independent variable. We examined the differences between the cluster size pre- and post-city lockdown using the Student’s two-sample *t*-test.

We calculated SAR as the proportion of RT-PCR test-positive contacts out of the total number of close contacts (including both test-negative and test-positive contacts), which was widely used to quantify the transmission risk among individuals with exposure history to sources of infection [29]. To characterise the heterogeneity that arose in the individual SAR, we assumed that the number of secondary cases (test-positive contacts) out of the total number of close contacts of an infector followed a beta-binomial distribution, where the probability of transmission (i.e. SAR) was assumed to be a random variable drawn from a beta distribution [30] (Appendix S5 in the **Online Supplementary Document**). We performed estimations of transmission heterogeneity by including all cases and stratifying by the infectors’ age (i.e. ages of 0-17, 18-65, and >65), vaccination status (i.e. number of doses received), contact settings, and epidemic period (i.e. pre- and post-city-wide lockdown).

To estimate the heterogeneity in the secondary transmissions, following Lloyd-Smith et al. [19], we fitted the identified case cluster data to a negative binomial distribution that was parametrised by the effective reproduction number *R* (mean parameter) and the dispersion parameter *k*. The lower the *k* value is, the higher the heterogeneity in the transmission. Terminal cases (i.e. cases that were identified as the end nodes of transmission chain or cluster) and sporadic cases were considered as those cases having zero offspring cases. Using the estimated *R* and *k*, we computed the expected proportion of cases that seeded 80% of transmission events [31,32] and the expected proportion of cases generating zero secondary cases (Appendix S4 in the **Online Supplementary Document**).

We estimated the model parameters with a Bayesian statistical framework by using the Markov chain Monte Carlo (MCMC) method (Appendix S4.3 and S5.2 in the **Online Supplementary Document**). We performed all statistical analyses in R (version 4.1.3) [33].

## RESULTS

From 7 August to 7 September 2022, 1139 COVID-19 cases infected with the BA.5.2 variants were reported in Urumqi, among which 43.0% were sporadic and 57.0% were epidemiologically linked with other cases (**Figure 1**, panel A). Among these, 1003 (88.1%) were vaccinated with at least one dose of inactivated vaccine before being test-positive, with 23 receiving one dose, 271 receiving two doses, and 709 receiving three doses. The process for collecting the data from the close contacts of the confirmed cases is shown in Appendix S3 in the **Online Supplementary Document**. We identified 51 786 close contacts that were linked with 769 seed cases. Among the 51 786 close contacts, 33 076 (63.9%) had a contact history with female cases, 48 746 (94.1%) with asymptomatic cases, and 46 805 (90.4%) with cases aged 18-65. Compared with the pre-city lockdown epidemic phase, the number of index cases increased, whereas the number of close contacts decreased (**Table 1**). The number of close contacts in various contact settings decreased consistently and substantially following the city lockdown on 10 August 2022 (**Figure 1**, panel B). The decreasing trend for the number of close contacts per index case within different contact settings and the difference in the mean size of case clusters between epidemic phases were statistically significant (**Figure 1**, panels C and 1D).

**Table 1:** Baseline characteristics of index cases infected with SARS-CoV-2 Omicron BA.5.2 variants and summary statistics of their close contacts

Characteristic of index case	Index case, n (%)	Number of contacts stratified by contact settings, n (SD)				
**Household**	**Community**	**Workplace**	**Unknown setting**	**Overall**
**Sex**						
Male	510 (44.8%)	695 (2.8)	398 (3.4)	778 (17.0)	16 839 (86.8)	18 710 (91.1)
Female	629 (55.2%)	965 (2.8)	1600 (16.0)	988 (7.7)	29 523 (158.5)	33 076 (165.4)
**Age**						
0-17	226 (19.8%)	234 (2.0)	141 (3.8)	16 (0.3)	3051 (31.4)	3442 (33.7)
18-65	821 (72.1%)	1317 (2.9)	1532 (11.1)	1698 (14.9)	42 258 (152.4)	46 805 (158.9)
>65	92 (8.1%)	109 (3.7)	325 (26.2)	52 (2.8)	1053 (26.0)	1539 (37.8)
**Symptomatic status**						
Symptomatic	83 (7.3%)	59 (1.3)	6 (0.4)	12 (0.9)	2963 (129.8)	3040 (131.3)
Asymptomatic	1056 (92.7%)	1601 (2.9)	1992 (12.6)	1754 (13.2)	43 399 (131.6)	48 746 (137.9)
**Vaccine dosage**						
0-1	159 (14.0%)	180 (3.1)	126 (4.3)	85 (3.7)	3597 (72.1)	3988 (77.7)
2	271 (23.8%)	330 (2.6)	477 (15.6)	111 (3.4)	5999 (78.7)	6917 (82.9)
3	709 (62.2%)	1150 (2.9)	1395 (11.8)	1570 (15.9)	36 766 (154.7)	40 881 (161.2)
**Epidemic period**						
Pre-lockdown	376 (33.0%)	744 (2.7)	1569 (19.5)	1366 (21.7)	26 407 (193.7)	30 086 (202.9)
Post-lockdown	763 (67.0%)	916 (2.9)	429 (5.4)	400 (2.7)	19 955 (81.8)	21 700 (83.8)

SD – standard deviation

**Figure 2:**
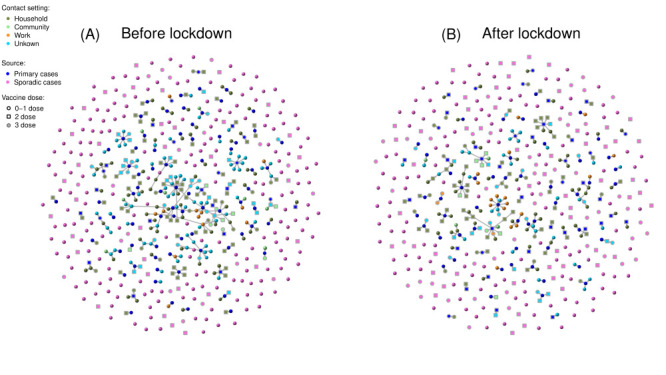
Visualization of the transmission network of all COVID-19 case clusters in Urumqi between August 7 and September 10, 2022. Epidemiologically linked cases are connected by grey edges, and the arrows denote the direction of transmission, stratified by the epidemic period. **Panel A.** Before city lockdown. **Panel B.** After city lockdown.

We collected contact tracing data for the 1139 laboratory-confirmed cases (649 linked cases and 490 sporadic cases) with Omicron BA.5.2 infection. After reconstruction of the epidemiological linkages between cases, we identified a total of 236 case clusters, 463 transmission pairs, and 186 transmission chains (**Figure 2**). Within the identified case clusters, 100 infectors generated at least two secondary cases. The largest case cluster involved 23 secondary cases directly seeded by a single case. Although the number of case clusters decreased, the transmission pattern remained similar pre- and post-city-wide lockdown, with most terminal transmissions (the end of the transmission chain or case clusters) occurring in households (**Figure 2**).

**Figure 3:**
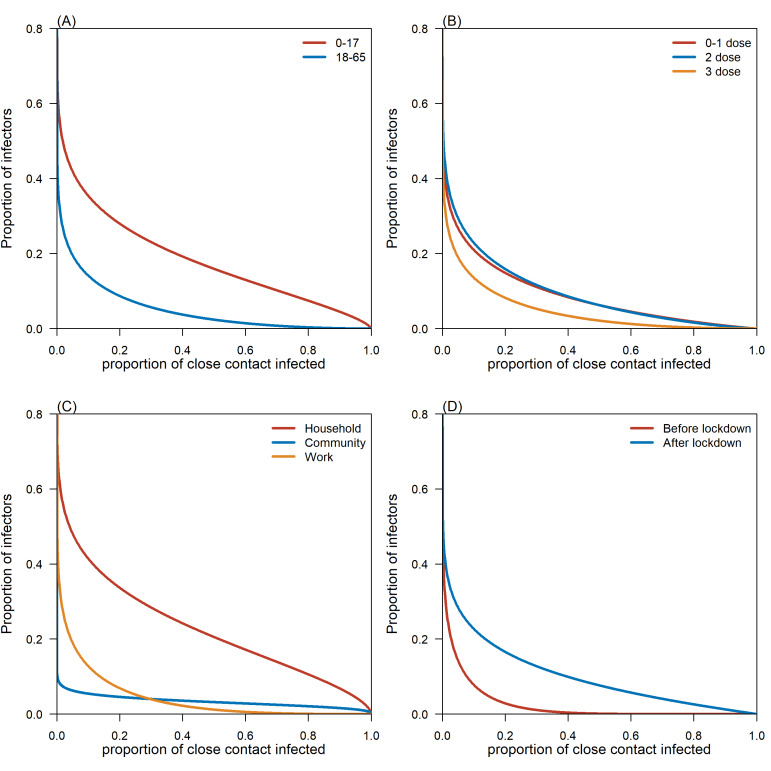
Complementary cumulative distribution function (i.e. tail distribution) of the fitted beta distributions of secondary attack rate (SAR). Each curve represents the proportion of seed cases (i.e. infectors) that had SAR larger than the given number on the horizontal axis. **Panel A.** Stratified by age groups. **Panel B.** Stratified by vaccine doses. **Panel C.** Stratified by contact settings. **Panel D.** Stratified by epidemic period.

After excluding 370 cases with no associated close contact, we included the remaining 769 index cases for estimating the SAR. The fitted beta-binomial distribution gave a mean SAR of 6.5% (95% credible interval (CrI) = 4.9-8.6) with a 95% percentile of 41% (95% CrI = 30-56) for all cases (Table S1 in the **Online Supplementary Document**). Female cases (8%, 95% CrI = 6-11), cases aged between 0-17 (14%, 95% CrI = 9-20), and symptomatic cases (10.4%, 95% CrI = 4.9-21.0) had relatively higher mean SAR estimates. The mean SAR decreased as the doses of vaccine that infectors received increased. Moreover, the mean SAR was the highest within household contact settings (21%, 95% CrI = 18-24) and was higher during the epidemic phase after the lockdown was imposed. A considerable proportion of infectors (approximately 20%) aged between 0-17 or with fewer than three doses of vaccine had an SAR larger than 20% (**Figure 3**, panels A and B). Furthermore, the SAR for household contact settings was estimated at above 40% for approximately 20% of infectors (**Figure 3**, panel C).

**Figure 4:**
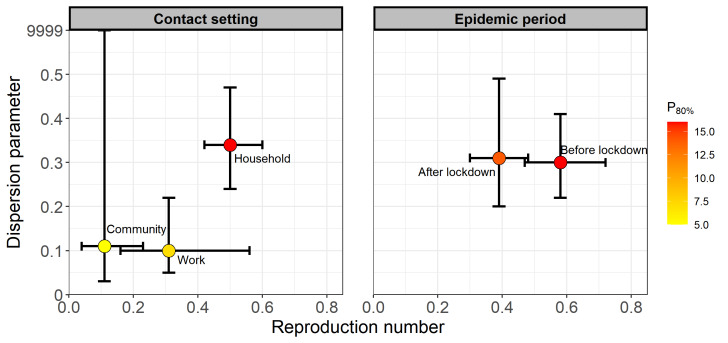
The estimated effective reproduction number (*R*) and dispersion parameter (*k*). The solid circles denote mean estimates, and the horizontal and vertical bars denote 95% CrIs of *R* and *k*, respectively. The gradient colour of middle dots denoted the proportion (%) of the most infectious cases that seeded 80% of transmissions. **Panel A.** Stratified by contact settings. **Panel B.** Stratified by epidemic period (right panel).

We estimated an *R* of 0.47 (95% CrI = 0.40-0.54) and a *k* of 0.27 (95% CrI = 0.21-0.34) across all contact settings (**Table 2**). Based on these estimates, we inferred that 14% (95% CrI = 12-17) of the cases with BA.5.2 infection seeded 80% of the local transmissions, with 76% (95% CrI = 72-80) of the cases leading to zero secondary infections (Figure S1 in the **Online Supplementary Document**). After fitting case clusters identified in different settings to the negative binomial distribution (**Table 2** and Figure S2 in the **Online Supplementary Document**), the estimated risk of transmission appeared to be the highest in the household setting, with an *R* estimated at 0.50 (95% CrI = 0.42-0.60), but was the lowest in the community settings, with an estimated *R* of 0.11 (95% CrI = 0.04-0.23). The degree of heterogeneity in the number of secondary infections was the highest in the community setting, with an estimated *k* of 0.11. The *R* estimates did not differ much across age groups and doses of vaccination, but the infectors aged 18-65 and those that had received a third dose of the vaccine had the largest heterogeneity in secondary transmission (**Table 2** and **Figure 4**). After the implementation of city lockdown, the *R* estimates decreased from 0.58 (95% CrI = 0.47 = 0.72) to 0.39 (95% CrI = 0.30-0.48), while the *k* estimates remained at the same level (**Table 2** and **Figure 4**).

**Table 2:** Summary of the estimated reproductive number (*R*) and dispersion parameter (*k*) of negative binomial distributions, stratified by different contact settings, age groups, vaccine doses, and epidemic periods

Stratifications	R (95% CrI)	k (95% CrI)	Prop80% (95% CrI)†	Prop0% (95% CrI)‡
Overall (n = 1139)*	0.47 (0.40-0.54)	0.27 (0.21-0.34)	14% (12-17)	76% (72-80)
**Type of contact setting**§				
Household (n = 515)	0.50 (0.42-0.60)	0.34 (0.24-0.47)	16% (13-20)	73% (68-78)
Community (n = 196)	0.11 (0.04-0.23)	0.11 (0.03-Inf)	5% (2-16)	93% (79-98)
Workplace (n = 203)	0.31 (0.16-0.56)	0.10 (0.05-0.22)	7% (4-13)	87% (76-93)
Unknown (n = 689)	0.33 (0.24-0.45)	0.16 (0.11-0.24)	10% (7-13)	83% (76-88)
**Age of infectors**				
0-17 (n = 226)	0.49 (0.35-0.67)	0.36 (0.21-0.64)	17% (12-24)	74% (62-82)
18-65 (n = 821)	0.46 (0.39-0.55)	0.24 (0.19-0.32)	14% (11-16)	77% (73-81)
>65 (n = 92)	0.49 (0.30-0.75)	0.55 (0.24-2.82)	20% (12-34)	70% (51-82)
**Vaccine doses of infectors**				
0-1 (n = 159)	0.48 (0.31-0.73)	0.46 (0.21-1.42)	19% (11-30)	72% (56-83)
2 (n = 271)	0.53 (0.39-0.71)	0.42 (0.25-0.80)	18% (13-26)	71% (60-79)
3 (n = 709)	0.45 (0.38-0.54)	0.22 (0.17-0.30)	13% (11-16)	78% (73-82)
**Epidemic period**				
Pre-lockdown (n = 376)	0.58 (0.47-0.72)	0.30 (0.22-0.41)	16% (13-20)	72% (66-78)
Post-lockdown (n = 763)	0.39 (0.30-0.48)	0.31 (0.20-0.49)	14% (11-19)	78% (71-83)

CrI – credible interval

*Includes the sporadic and terminal cases, i.e. sample size.

†Expected proportion of the most infectious seed cases that were responsible for 80% of transmission events.

‡The expected proportion of seed cases with 0 secondary case, i.e. no transmission.

§The summation of sample sizes in different contact settings was larger than the overall sample size (515 + 196 + 203 + 689 > 1139) because some index cases had close contacts (and possibly offspring cases) in more than one contact settings, and thus the transmission of such index cases in different contact settings were accounted separately. Besides, for each contact setting, the sample size was smaller than the overall sample size (515 < 1139, 196 < 1139, 203 < 1139, and 689 < 1139. This was because an index case would not be counted in a contact setting, if this index case has 0 close contact in this contact setting.

## DISCUSSION

Since December 2021, the Omicron variant of SARS-CoV-2 has rapidly spread around the world and has constantly been evolving. The unique study population (i.e. high vaccination coverage but almost no history of natural infections) and public management measures policy (i.e. frequent mass SARS-CoV-2 testing of the entire city with proactive contact tracing of every infected case) created a unique research site for this study, allowing us to study the essential characteristics of SARS-CoV-2 Omicron transmission, including the heterogeneity of transmission and superspreading potentials. Using detailed contact tracing data collected during the outbreak in Urumqi, China, we investigated the heterogeneity in the transmission risk of the Omicron BA.5.2 sub-lineage across demographic and clinical factors of cases, contact settings, and epidemic phases. Broadly, the risk of transmission was lower for male cases, middle-aged cases, asymptomatic cases, and those that received a full course of vaccine than for their within-group counterparts. Among contact settings, BA.5.2 was more transmissible in households than in workplace and community settings. The imposition of city lockdown was also the risk modifier of the transmission and superspreading potentials of COVID-19.

Other studies also found higher transmission risks (both SAR and *R*) within households compared with other settings [23,24,34], possibly because they often featured an enclosed area, prolonged exposures, and lack of protection with face masks. The higher risk of transmission within households could also contribute to the increment in the SAR estimates after the city-wide lockdown, as most of the non-household contacts were curbed and within-household contacts increased due to the physical confinement. Therefore, effective measures to reduce within- and between-household transmission, such as rapid identification and quarantine of household contacts, should be considered in future outbreaks [35]. We found that the transmission within community settings (e.g. health care givers and patients in the same ward, persons sharing a vehicle or restaurant, and community workers having contact with cases in public places) was much more dispersed, with the lowest SAR, *R*, and *k* estimates among all contact settings, which was consistent with the findings for the wild-type strain from a previous study [34]. The rapidly implemented lockdown measures likely contribute largely to the lower values of SAR and *R* across the community settings. Although a lower *k* value represented a higher potential of observing superspreading events in some circumstances [11,17,36], the excessive “class zero” (i.e. the proportion of cases leading to zero secondary infections) was the hallmark of a negative binomial offspring distribution with sufficiently low *R* and *k* values [18] (e.g. *R* = 0.11 and *k* = 0.11 in the community setting), under which the probability of large outbreaks is extremely low. Nonetheless, outbreaks that emerged from highly heterogeneous transmission dynamics with low *k* values were more likely to be explosive with shorter doubling times and higher epidemic peaks [18,21].

Although Omicron BA.5 variants were characterised by a stronger ability to escape the immune response, we found that the third (i.e. booster) dose of vaccination might be associated with some protection against BA.5.2 transmission (Table S1 in the **Online Supplementary Document**). Moreover, we found that the virus was more transmissible among younger cases than adult cases during the BA.5.2 outbreaks, likely due to the lower vaccination coverage among younger generations compared with older generations in Urumqi (70.28% versus 79.28%). We also found that female cases had a higher transmission risk than male cases, possibly due to the them having a higher frequency of social contact [37]. Although the lockdown decreased the average *R-*value, the heterogeneity in *k*-value remained unaffected. This is in line with the findings of Lloyd-Smith et al. [18], indicating that population-wide public health interventions are effective in reducing population infectiousness but have no effect on transmission heterogeneity. They found that, compared with population-wide control measures, individual-specific measures (e.g. targeting high-risk settings and super-spreaders) would lead to more efficient mitigations by reducing the effective reproduction number but raising the heterogeneity, which favours disease extinction [18]. Thus, targeted PHSMs that recognise the differential risk of transmission between contact settings and demographic factors should be prioritised when planning control policies.

As a measure of transmission probability per contact, SAR was always treated as an average characteristic across the whole case population or contact settings [24,38,39]. We fitted the contact tracing data to the beta-binomial distribution, assuming SAR to be a random variable following a beta distribution. The estimated SAR of all Omicron BA.5.2 cases was higher than that of the wild-type strain (<4%) [19,24] and the previous circulating Delta VOC (1.4%) [40], indicating the higher transmissibility of the novel variant among populations with high vaccine coverage. The higher variance-to-mean ratio of the estimated SAR distributions across groups suggested a significant heterogeneity in the probability of transmission for the Omicrion BA.5.2 outbreak. Specifically, the variance to mean ratio of SAR in community and workplaces was 2.64- and 1.80-fold higher than in the household settings, respectively, which might be due to the relatively low variance in the number of households and contacts.

Our study has some limitations. First, our analysis depends on the accuracy of the contact tracing data, which was subject to recall bias and underreporting of cases. However, the case detection rate was assumed to be higher during the study period in Urumqi, given that the local outbreaks were quickly contained by intensive outbreak investigation, contact tracing, and mass testing. Second, the contact setting information for a large proportion of close contacts was not available, which may influence our estimated transmission heterogeneity between contact settings (e.g. the less conclusive *k* estimates within community settings). Third, our contribution to the understanding of the individual heterogeneity in SAR should be interpreted in the context of stringent PHSMs, under which the individual level of exposure was truncated due to the shortened infectious period of infectors such that the intrinsic SAR of BA.5.2 variants would be higher than what we obtained here. Previous studies explicitly modelled the individual heterogeneity of exposure in the SAR, and the resultant estimates could be more generalisable [19,23,41]. Additionally, we did not consider the heterogeneity in the infectious period. Previous studies explicitly modelled the individual variations in the infectious period and demonstrated that this source of heterogeneity could also shape the outbreak dynamics [21]. Modelling studies are warranted to further investigate the impact of variations in SAR on transmission dynamics.

## CONCLUSIONS

We found that, in the context of intensive control measures, active case detection, and relatively high vaccine coverage, but with an infection-naive population, the contact pattern and transmission risks of cases infected with Omicron BA.5variants manifested substantial heterogeneity across different demographic strata, vaccine statuses, and contact settings. Given the rapid evolution of SARS-CoV-2, investigating the distribution of transmission could not only help to promote public awareness and preparedness among high-risk groups, but also stress the importance of continuously monitoring the transmission characteristics of genetic variants of SARS-CoV-2.

## Additional material


Online Supplementary Document
