# Supplementary material for: Case clustering, contact stratification, and transmission heterogeneity of SARS-CoV-2 Omicron BA.5 variants in Urumqi, China: An observational study
Source: J Glob Health. 2023 May 19;13:06018. doi: 10.7189/jogh.13.06018 (PMC10193892; doi:10.7189/jogh.13.06018)
Supplement: Online Supplementary Document [file jogh-13-06018-s001.pdf]

Appendix

Table of Contents

S1 Definitions of COVID-19 case, close contact, transmission pair, and case cluster 2
S2 Confirmation of genetic sequence 3
S3 Data cleaning 4
S4 Modelling secondary cases distribution and superspreading 5
S4.1 Secondary case distribution 5
S4.2 Expected proportion of seed cases generating 80% of transmission 5
S4.3 Parameter estimation 5
S4.4 Supplementary results 6
S5 Modelling heterogeneity in transmission risk 8
S5.1 Distribution of secondary attack ratio 8
S5.2 Parameter estimation 8
S5.3 Supplementary results 8
Supplementary references 10

S1 Definitions of COVID-19 case, close contact, transmission pair, and case cluster

All COVID-19 cases were defined as individuals who received a positive test outcome (with Ct value < 40) from real-time reverse transcription polymerase chain reaction (RT-PCR) assay for the genetic segments of SARS-CoV-2 strains using ORF1ab gene or N gene detection kit.

The definition of clinical severity after SARS-CoV-2 infection was as follows. For a symptomatic case, it was defined as a case presenting one of the relevant clinical symptoms, including fever, respiratory symptoms, and radiographic evidence of pneumonia. The asymptomatic or mildly symptomatic SARS-CoV-2 infection, which was classified as "asymptomatic" in this study, was defined as a case presenting one of the relevant clinical symptoms for less than 7 days, including fever, or respiratory symptoms, and without a radiographic evidence of pneumonia. For asymptomatic infection, it was defined as a case having no clinically evident symptoms. Since most of confirmed cases (92.7%) were asymptomatic, we avoided to further classify the clinical severity of cases into more detailed levels due to limited samples.

We defined the close contacts of a confirmed COVID-19 case as individuals having an epidemiologic link to a COVID-19 case, i.e., individuals free from symptoms and COVID-19 diagnosis exposed to a RT-PCR test positive person. As a considerable amount of transmission could occur at very early stage after infection [1, 2], individuals who had been exposed to a case within 4 days before the test-positive date of the case would also be counted as close contacts. We classified the close contacts of confirmed cases into categories described as follows:

- household contacts (i.e., household members regularly living within the same or close space, or relatives who had close contact with case)
- workplace or school contacts (i.e., a work colleague or classmate), and
- community contacts (i.e., healthcare-givers and patients in the same ward, persons sharing a vehicle or restaurant, and community workers having contact with case in public places).
- unknown contacts (i.e., only show for the contact with the space, no specific contact way).

For those contacts who were (eventually) test-positive for COVID-19, we treated these contacts as infectee, and their source case (who were confirmed with COVID-19 in the first place) as infector and forms transmission pairs.

Based on the identified transmission pairs, we thereafter grouped the linked cases into case-clusters, which is defined as a case or a cluster of cases (i.e., infectees) with a common single source of infection (i.e., infectors). The number of secondary cases generated by each infector was then extracted. The number of secondary cases generated by each infector was then extracted. As there might be epidemiological linkages between case clusters, we further linked those case-clusters into transmission chains, which could involve multiple generation of infections. Based on the locations where the infection occurred, we also identified 3 contact settings, including household, community, and workplace. The case clusters and transmission chains were constructed independently by 2 authors. The final list of included cases was decided following discussion between the authors, with full agreement required prior to inclusion.

S2 Confirmation of genetic sequence

Nasopharyngeal or oropharyngeal swabs specimen from 11 confirmed cases during the first few days of the outbreak were collected, and undergone whole-genome sequencing. MAFFT program was used to perform multiple sequence alignments, and the GTR + CAT nucleotide substitution model in FastTree (version 2.1.11) were applied to explore the phylogenetic relationship. On the basis of Phylogenetic Assignment of Named Global Outbreak (PANGO) lineage designation, the samples were eventually classified as SARS-CoV-2 Omicron BA.5.2 sub-lineage.

There was a total of 62 amino acid (AA) substitutions in different genetic segments of SARS-CoV-2, including 31 in spike (S) protein, 19 in non-structural proteins (NSPs) ORF1a and ORF1b, 4 in membrane protein, 4 in nucleocapsid (N) protein, 3 in auxiliary proteins ORF3a and ORF9b, and 1 in envelope (E) protein.

S3 Data cleaning

We collected epidemiological contact tracing data of laboratory-confirmed cases with Omicron BA.5.2 infection between August 7 and September 7, 2022, from the Xinjiang Uygur Autonomous Region Health Committee. A total of 1139 confirmed cases were included. Among these confirmed cases, 649 were those within case cluster with size >1, and 236 were infector with >0 offspring cases, 413 terminal cases. There were 490 sporadic or cases with unknown source (i.e., cases without known source of infection and secondary cases). Of the 1139 positive cases, 370 test-positive individuals during isolation with 0 close contact, and the remaining 769 test-positive individuals associating with 51323 test-negative close contacts. Among these contacts, according to the classification of places of contact (**Appendix S1**), there were 1660 household contacts, 1998 community contacts, 1766 workplace contacts and 46362 unknown contacts.

S4 Modelling secondary cases distribution and superspreading

S4.1 Secondary case distribution

Given the stochastic effect of the transmission events, we assumed the number of secondary cases generated by an infector followed a Negative binomial (NB) distribution which was parametrized by a reproduction number (*R*) and a dispersion parameter (*k*), as followed by previous studies [3,4]. The probability mass function of NB distribution is given by:

$$\Pr(Z = z; R, k) = \frac{\Gamma(k+z)}{\Gamma(k)\cdot\Gamma(z+1)}\left(\frac{R}{R+k}\right)^{z}\left(\frac{k}{R+k}\right)^{k}$$

Here, *z* is the number of secondary cases generated by an infector, and $\Gamma()$ denotes the gamma function.

S4.2 Expected proportion of seed cases generating 80% of transmission

Given the *R* and *k* estimates, we calculated the expected proportion of cases that were responsible for 80% of all transmissions [3], which is given by [4]:

$$1 - P = \int_0^Z \Pr(Z = z; R, k)\,\mathrm{d}z$$

where *Z* satisfies:

$$1 - 80\% = \frac{1}{R}\int_0^Z \lfloor z \rfloor \Pr(Z = z; R, k)\,\mathrm{d}z$$

Here, $\lfloor\cdot\rfloor$ denotes the floor function.

S4.3 Parameter estimation

The parameters of negative binomial distribution were estimated by using the Metropolis-Hastings algorithm with noninformative prior distributions, which is a Markov chain Monte Carlo (MCMC) method. The marginal posterior distribution was obtained from 50000 iterations, among which the first 10000 samples were discarded as for burn-in. The convergence of each MCMC chain was checked by using the trace plot and Gelman-Rubin-Brooks convergence diagnostic [5]. The median and the 95% credible interval (CrI) were obtained from the marginal posterior distributions.

We compared the fitting performance of negative binomial (NB) distribution to that of Poisson distribution (i.e., setting *k* as infinity) and that of Geometric distribution (i.e., setting $k = 1$) by using the deviance information criterion (DIC), and found that NB distribution had a relatively lower DIC value (data not shown).

S4.4 Supplementary results

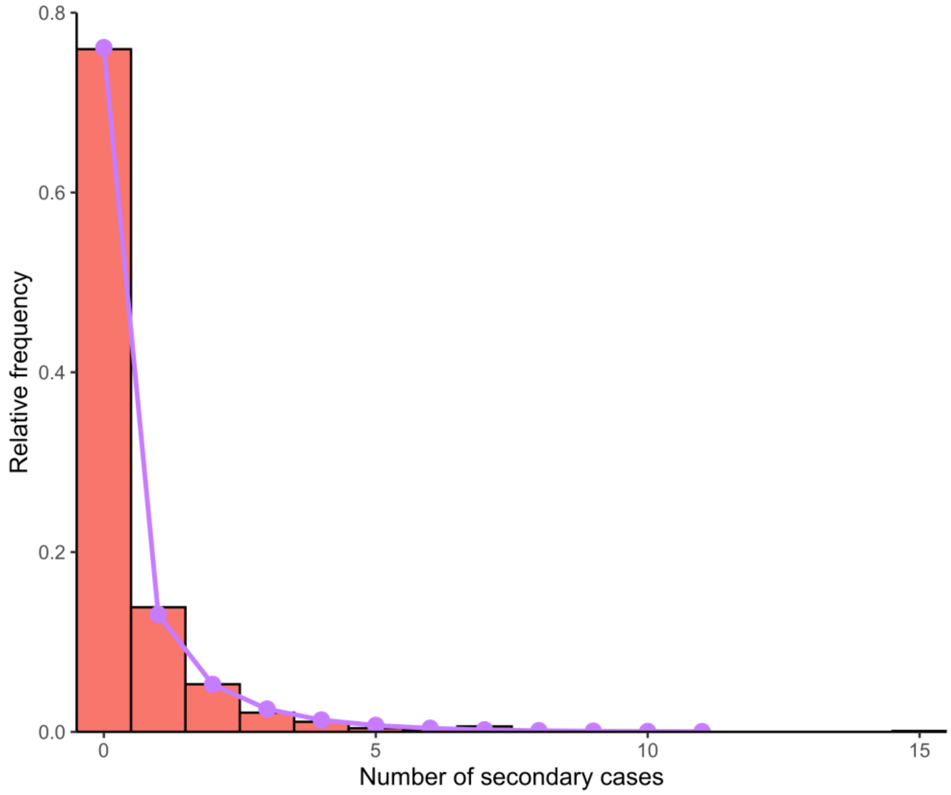

Figure S1. The observed secondary case distribution (red blocks), and the fitted negative binomial distribution (purple curve). The purple solid curve represented the median of MCMC posterior samples.

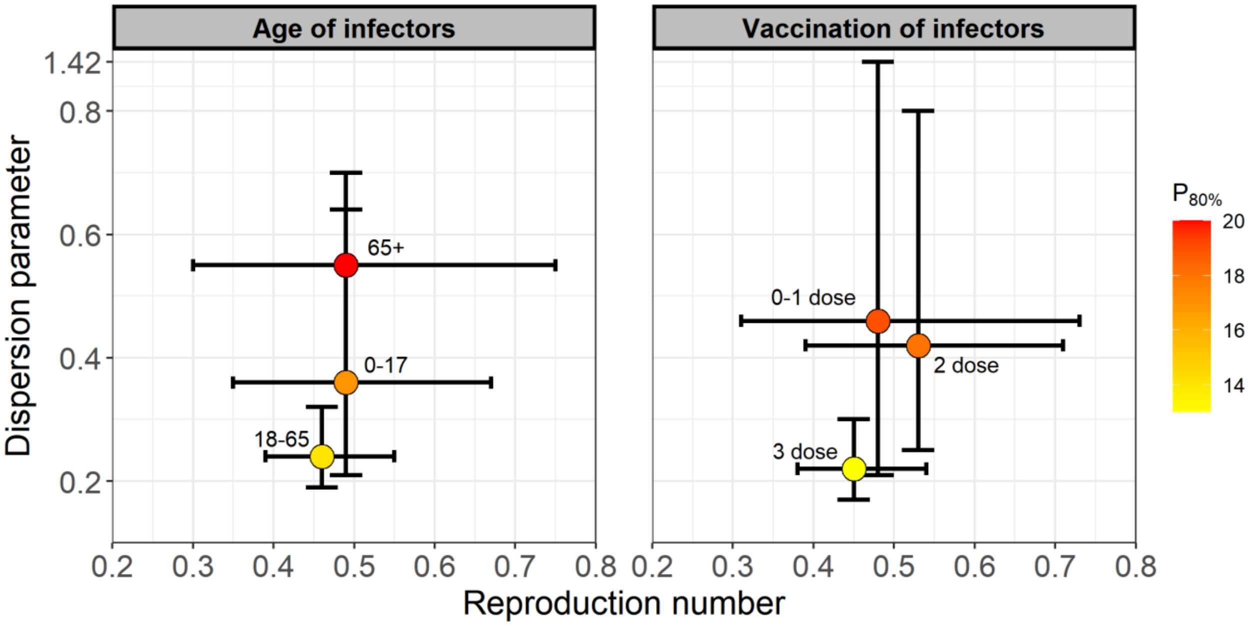

Figure S2. The estimated effective reproduction number (*R*) and dispersion parameter (*k*) stratified by age groups and vaccine doses. The solid circles denoted mean estimates and the horizontal and vertical error bars denoted 95% CrIs of *R* and *k*, respectively. The gradient color of middle dots denoted the proportion (%) of the most infectious cases that seeded 80% transmissions.

S5 Modelling heterogeneity in transmission risk

S5.1 Distribution of secondary attack ratio

The secondary attack ratio (SAR) is defined as the probability that infections occur among people who exposed to the infectors**.** We assumed the number of secondary cases k_i out of the total number close contacts n_i of an (randomly-selected) infector case i followed a binomial distribution, conditioning on the SAR, P_i. To take account of the individual variations in SAR, we assumed the P_i followed a Beta distribution that parametrized by two shape parameters α and β, and then the mean of P_i was $\frac{\alpha}{\alpha+\beta}$. This would thus yield a beta-binomial distribution for k_i. The probability mass function of the beta-binomial distribution for k_i is given by [6]:

$$f = \frac{\binom{n_i}{k_i}\mathrm{Be}(k_i+\alpha, n_i-k_i+\beta)}{\mathrm{Be}(\alpha,\beta)}$$

where x denotes the number of secondary cases and size denotes the number of close contacts, and $\mathrm{Be}(\alpha,\beta)=\int_0^1 x^{\alpha-1}(1-x)^{\beta-1}\,\mathrm{d}x$ the beta function (which is not Beta distribution) at α and β. We constructed the likelihood function over all cases as follows:

$$L_{\mathrm{SAR}} = \prod_{i=1}^{N}\frac{\binom{n_i}{k_i}\mathrm{Be}(k_i+\alpha, n_i-k_i+\beta)}{\mathrm{Be}(\alpha,\beta)}$$

Here, parameters α and β for the beta distribution were to be estimated, but the counts of k_i test-positive contacts out of a total number of close contacts n_i were both known from real-world observations.

S5.2 Parameter estimation

The parameters of beta-binomial distribution were estimated in a Bayesian statistical framework by applying the Markov chain Monte Carlo (MCMC) method. The Metropolis-Hastings algorithm with noninformative prior distributions were used, each marginal posterior distributions was obtained from 50000 iterations, among which the first 10000 samples were discarded as for burn-in. The convergence of each MCMC chain was checked by using the trace plot and Gelman-Rubin-Brooks convergence diagnostic [5]. The median and the 95% credible interval (CrI) were obtained from the marginal posterior distributions.

We compared the fitting performance of beta-binomial distribution to that of binomial distribution (i.e., treating SAR as a constant among source cases) by using the deviance information criterion (DIC), and found that beta-binomial distribution had a relatively lower DIC value (data not shown).

S5.3 Supplementary results

Table S1. Summary of the estimated secondary attack ratio (SAR) stratified by age groups, vaccine doses, contact settings, and epidemic period.

Stratifications	Sample size	Mean (%)	SD (%)	95% percentile (%)
**Overall**[#]	769	6.5 (4.9, 8.6)	15.0 (12.0, 19.0)	41.0 (30.0, 56.0)
**Sex**				
Male	313	4.3 (3.2, 6.1)	9.1 (6.8, 13.0)	24.0 (16.0, 36.0)
Female	456	8.2 (6.0, 11.0)	19.0 (15.0, 24.0)	57.0 (39.0, 78.0)
**Age**				
0-17	122	14.0 (9.0, 20.0)	26.0 (18.0, 33.0)	82.0 (54.0, 97.0)
18-65	599	5.0 (3.8, 7.0)	13.0 (10.0, 16.0)	33.0 (23.0, 45.0)
>65[*]	48	9.1 (3.1, 17.0)	24.0 (6.0, 33.0)	NA
**Type of index cases**				
Symptomatic	44	10.4 (4.9, 21.0)	19.0 (9.5, 31.0)	57.0 (25.0, 95.0)
Asymptomatic	725	6.0 (4.2, 8.0)	14.0 (10.6, 18.0)	38.0 (26.0, 52.0)
**Vaccine dose of index cases**				
0-1	79	9.6 (5.4,17.0)	19.0 (12.0, 30.0)	58.0 (31.0, 93.0)
2	159	9.2 (5.3, 14.0)	19.0 (11.0, 26.0)	56.0 (29.0, 81.0)
3	531	5.0 (3.6, 7.0)	12.0 (9.0, 16.0)	32.0 (22.0, 45.0)
**Type of contact setting**[$]				
Household	515	21.0 (18.0, 24.0)	30.0 (27.0, 34.0)	92.0 (84.0, 97.0)
Community	196	5.3 (3.2, 8.4)	20.0 (14.0, 26.0)	50.0 (9.2, 98.0)
Workplace	203	3.5 (2.0, 6.2)	9.0 (4.0, 17.0)	21.0 (10.0, 44.0)
Unknown	689	2.2 (1.4, 3.4)	7.8 (5.0, 12.0)	14.0 (8.2, 24.0)
**Epidemic period**				
Before lockdown	317	2.0 (1.8, 3.9)	6.2 (4.1, 9.8)	15.0 (9.6, 23.0)
After lockdown	452	9.8 (7.0, 13.0)	21.0 (16.0, 24.0)	63.0 (44.0, 78.0)

# The sample size of here was calculated as (1139 – 370 =) 769 index cases, where 1139 was the total number of confirmed COVID-19 cases and 370 was the number of index cases with 0 associated contacts (0 contact). Those 370 index cases were excluded from statistical analyses of SAR.

* The estimates of posterior MCMC samples did not converge, which might be due to a relatively small sample size, and thus the mean and SD were summarized as the sample mean and sample SD, respectively, with 1000 runs of bootstrap.

$ The summation of sample sizes in different contact settings was larger than the overall sample size, i.e., 515 + 196 + 203 + 689 > 769. This was because some index cases had close contacts in more than one contact settings, and thus the SARs of such index cases were calculated separately. Besides, for each contact setting, the sample size was smaller than the overall sample size, i.e., 515 < 769, 196 < 769, 203 < 769, and 689 < 769. This was because an index case would not be counted in a contact setting, if this index case has 0 close contact in this contact setting.

Supplementary references

1. He X, Lau EHY, Wu P, Deng X, Wang J, Hao X, Lau YC, Wong JY, Guan Y, Tan X *et al*: **Temporal dynamics in viral shedding and transmissibility of COVID-19**. *Nature Medicine* 2020, **26**(5).
2. Hu S, Wang W, Wang Y, Litvinova M, Luo K, Ren L, Sun Q, Chen X, Zeng G, Li J *et al*: **Infectivity, susceptibility, and risk factors associated with SARS-CoV-2 transmission under intensive contact tracing in Hunan, China**. *Nat Commun* 2021, **12**(1):1533.
3. Lloyd-Smith JO, Schreiber SJ, Kopp PE, Getz WM: **Superspreading and the effect of individual variation on disease emergence**. *Nature* 2005, **438**(7066):355-359.
4. Endo A, Abbott S, Kucharski AJ, Funk S, Centre for the Mathematical Modelling of Infectious Diseases C, Working G: **Estimating the overdispersion in COVID-19 transmission using outbreak sizes outside China**. *Wellcome open research* 2020, **5**:67-67.
5. Gelman A, Carlin JB, Stern HS, Dunson DB, Rubin DB: **Bayesian data analysis, third edition**. *Journal of the American Statistical Association* 2003, **45**(2).
6. Prentice RL: **Binary Regression Using an Extended Beta-Binomial Distribution, with Discussion of Correlation Induced by Covariate Measurement Errors**. *Journal of the American Statistical Association* 1986, **81**(394):321-327.
